# The Bacillus Tuberculosis and the Refutation of Its Etiological Relation with Tuberculosis

**Published:** 1883-01

**Authors:** 


### SELECTIONS.

### The Bacillus Tuberculosis and the Refutation of its Etiological Relation with Tuberculosis.

In a paper on this subject Dr. H. F. Formad, of the University of Philadelphia, said:

It seems to me that there are serious errors in Koch's work, and that he has overlooked anatomical facts and pathological laws in immediate connection with it, and has thus been led to misinterpretation of his own results. He could not have been aware of this, as it is not probable that he purposely would ignore established facts.

Before pointing out the deficiency of Koch's proofs of, and arguments for, the infectiousness of phthisis, and for the existence of a specific organism, the Bacillus tuberculosis,·I beg leave to announce briefly the main facts of my researches on the minute structure of connective tissue in the so-called scrofulous persons and animals. I have devoted over four years to this special study, making my experiments on animals.

My researches clearly show the following points:

1st. The predisposition to tuberculosis in some men and animals, the so-called scrofulous habit, lies in the anatomy of the connective tissue of the individual, the peculiarity being a narrowness of the lymph-spaces, and their partial obliteration by cellular elements.

2d. Only beings with such anomalous structure of connective tissue can have primary tuberculosis, and such animals invariably do become tuberculous from any injury resulting in inflammation, or from repeated injuries.

3d. Scrofulous beings can have no other than a tuberculous inflammation, although it may remain local and harmless.

4th. Non-scrofulous men or animals may acquire the predisposition to tuberculosis through malnutrition and confinement, the latter bringing on the above-mentioned anatomical peculiarities in the connective tissue.

5th. No external etiological influences are necessary to cause tubercular disease other than those which ordinarily produce inflammation, and even scrofulous beings will not become tuberculous unless local inflammation is set up. No inflammation, no tuberculosis.

6th. Non-scrofulous animals, so far as can be established now, may acquire tubercular disease through injuries of serious membranes, viz.; peritoneum, pleura, etc., and even here without any special virus whatsoever. Clinical observations on the post-mortem table show similar conditions and prove the same in man. (Koch's own experiments are also in favor of this proposition, as will be shown hereafter; but he has overlooked this.)

7th. The bacilli, which it is the merit of Koch to have first proved to infest the tissues affected by tuberculous disease, are not necessary for its causation, even if a special organism exist and be really possessed of such property. The presence of bacilli (so far as our present research goes) is secondary, and appears to *condition* the complete destruction of the tissue already diseased and infested by them, and this destruction is in direct proportion to the quantity of organisms, which thus regulate the prognosis. The tubercular tissue seems to serve merely as a nidus for the growth of the bacillus.

8th. From the results of microscopic examination, form numerous observations upon the post-mortem table, and on clinical grounds, I have come to the conclusion that phthisis is not a specific infectuous disease, but that the individuals suffering from tubercular disease are specific themselves originally, and form a special species of mankind, the "scrofulous."

9th. Scrofulosis is a condition which may arise from malnutrition and seclusion in any being, and thus may be produced artificially. It always depends upon the demonstrated anatomical changes in the connective tissue.

10th. An analysis of Koch's experiments shows that he has not proved the parasitic nature of phthisis, or that there exists a special Bacillus tuberculosis; so that the infectuousness of tubercular disease is still *sub judice.*

